# Universal domain wall dynamics under electric field in Ta/CoFeB/MgO devices with perpendicular anisotropy

**DOI:** 10.1038/ncomms13532

**Published:** 2016-11-16

**Authors:** Weiwei Lin, Nicolas Vernier, Guillaume Agnus, Karin Garcia, Berthold Ocker, Weisheng Zhao, Eric E. Fullerton, Dafiné Ravelosona

**Affiliations:** 1 Center for Nanoscience and Nanotechnology, Université Paris-Sud–CNRS, UMR 9001, 91405 Orsay, France; 2 Singulus Technologies AG, 63796 Kahl am Main, Germany; 3 Center for Memory and Recording Research, University of California San Diego, La Jolla, California 92093, USA

## Abstract

Electric field effects in ferromagnetic metal/dielectric structures provide a new route to control domain wall dynamics with low-power dissipation. However, electric field effects on domain wall velocities have only been observed so far in the creep regime where domain wall velocities are low due to strong interactions with pinning sites. Here we show gate voltage modulation of domain wall velocities ranging from the creep to the flow regime in Ta/Co_40_Fe_40_B_20_/MgO/TiO_2_ structures with perpendicular magnetic anisotropy. We demonstrate a universal description of the role of applied electric fields in the various pinning-dependent regimes by taking into account an effective magnetic field being linear with the electric field. In addition, the electric field effect is found to change sign in the Walker regime. Our results are consistent with voltage-induced modification of magnetic anisotropy. Our work opens new opportunities for the study and optimization of electric field effect at ferromagnetic metal/insulator interfaces.

## Abstract (web summary)

Domain walls in ferromagnetic–oxide structures can be moved using an electric field, which could be useful for low-power electronic devices. Here, the authors demonstrate the modulation of the velocity of these domain walls between a creep and a flow regime.

## Graphical abstract

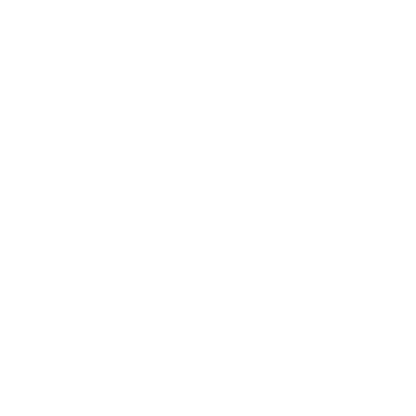

Electric field control of magnetic domain wall (DW) motion in transition-metal ferromagnets^1^,^2^,^3^,^4^,^5^,^6^,^7^ has attracted great interest due to the possibility to achieve DW control with low-power dissipation for memory and logic circuits. One important example is racetrack memory^8^, where a gate voltage can be exploited to assist manipulation of DWs under spin-polarized currents^1^. This would allow the reduction of the generally large critical currents needed to depin and shift DWs in narrow wires. Another example concerns DW logic circuits^9^, for which local modulation of DW motion can lead to programmable DW energy landscapes. In both cases, the key is whether the electric field enables both efficient depinning for stored DWs and control of DW propagation.

Materials with perpendicular magnetic anisotropy (PMA) are particularly attractive for DW devices as they exhibit narrow DWs^10^, enabling high-density storage. Recently, the electric field effect on DW dynamics has been demonstrated in Pt/Co/oxide films with PMA involving oxides with relatively high dielectric constant such as AlO_*x*_ (refs 3, 6), HfO_2_ (ref. 4) or GdO_*x*_ (ref. 5). However, significant modulation of DW velocity under gate voltage has been shown only in the creep regime where DWs propagate at relatively low speeds (<0.1 m s^−1^) through thermal activation over local energy barriers^3^,^4^,^5^,^6^ originating from the random disorder^10^,^11^. The efficient gate-voltage control in the creep regime has been explained in terms of modulation of the activation energy barriers^3^,^4^,^5^,^6^ through voltage-induced changes of the interfacial magnetic anisotropy^12^,^13^,^14^,^15^,^16^. With increasing DW velocity in the creep regime, the electric field effect was found to be strongly reduced due to the decrease of the activation energies with applied magnetic fields^5^. So far, there has been no reports on the experimental observation of electric field effects beyond the creep regime, particularly in the depinning and flow regimes^17^,^18^,^19^,^20^.

Here we report the observation of voltage modulation of DW motion over different dynamical regimes where velocities range from 10^−8^ to 20 m s^−1^. In addition, we demonstrate that this behaviour can be understood over the full range of DW dynamics by electric field-induced modulation of the magnetic anisotropy. Our approach makes use of Ta/CoFeB/MgO films with PMA, for which the density of pinning sites and the damping are very low^21^,^22^,^23^ with respect to other PMA materials such as Co/Pt^19^,^24^ or Co/Ni^18^,^25^. In addition, PMA at the CoFeB/MgO interface can be manipulated efficiently through gate voltage control^15^,^16^. These materials are considered as the most promising, not only for spin transfer torque magnetic random access memory^26^,^27^ but also for DW-based memories^28^, as a combination of spin Hall effect and Dzyaloshinskii–Moriya interaction leads to efficient DW propagation under current^29^,^30^,^31^.

## Results

### Voltage effect on DW velocity in the depinning regimes

**Figure 1:**
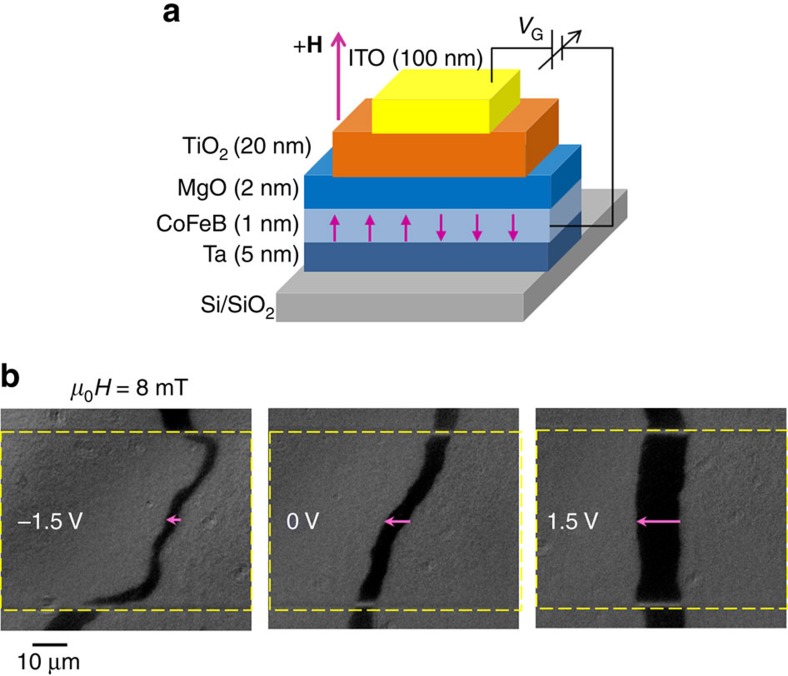
Schematic of the experiment and magnetic domain wall displacement under gate voltages in an annealed Ta/Co_40_Fe_40_B_20_/MgO/TiO_2_ structure. (**a**) The gate voltage *V*_G_ is applied between the ITO strip and the CoFeB film. The magnetic field **H** is applied perpendicular to the film plane. (**b**) DW displacement measured by polar Kerr microscopy at applied magnetic fields *μ*_0_*H* of 8 mT with 45 μs duration under voltage *V*_G_ of −1.5, 0 and 1.5 V, respectively. The DW displacement is obtained from the difference between the polar Kerr images after and before magnetic field pulses. The right (left) boundary in the dark part in the image shows the DW position before (after) applying the magnetic field pulse and the arrow indicates the direction of DW motion. Top gate voltage *V*_G_ is applied in the region indicated by the dashed rectangle.

**Figure 2:**
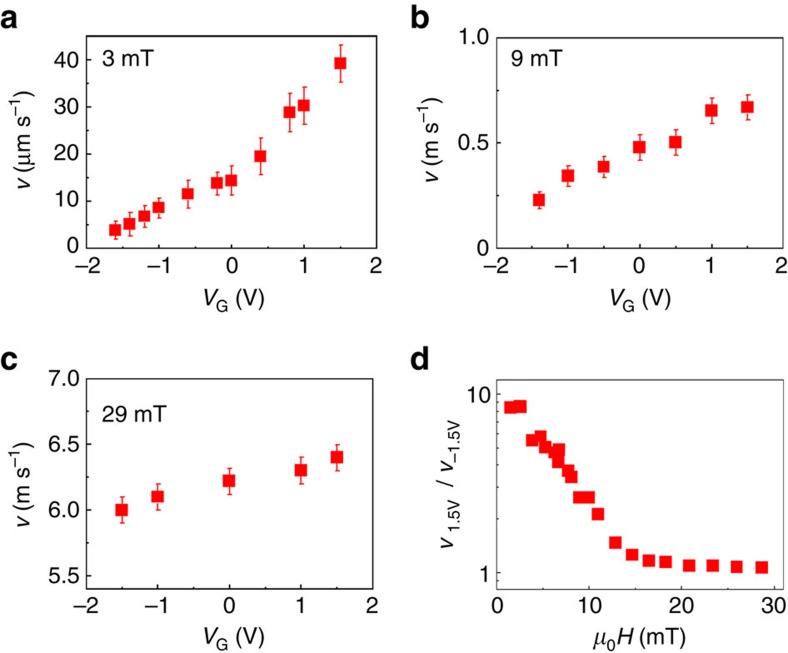
Magnetic DW velocity as a function of the voltage gate for different magnetic fields in the annealed Ta/CoFeB/MgO structure. (**a**–**c**) DW velocity as a function of *V*_G_ for different magnetic fields *μ*_0_*H* of 3, 9 and 29 mT, respectively. (**d**) Ratio of DW velocity *v*_1.5V_/*v*_−1.5V_ as a function of the applied field. Electric field effect is detected up to the high velocity regime. The error bars of DW velocity are given by repeating the measurements for several times in one device. As DW motion is intrinsically stochastic due to thermal activation over energy barriers, a distribution of DW velocity is expected.

In this study, DW velocity under electric field was measured in both as-grown and annealed Ta(5 nm)/Co_40_Fe_40_B_20_(1 nm)/MgO(2 nm)/TiO_2_(20 nm)/ITO samples using magneto-optic Kerr microscopy under combined electric and magnetic fields. By applying a top gate voltage *V*_G_ between the bottom Ta/CoFeB and top ITO electrodes (shown schematically in Fig. 1a), electrons can either accumulate or deplete at the CoFeB/MgO interface. For the experiments shown, positive (negative) voltage corresponds to electron accumulation (depletion) at the CoFeB/MgO interface. It is noteworthy that, to avoid any temperature rise, the experiments were performed under low leakage current below 20 nA, which corresponds to gate voltages ranging from −1.5 to 1.5 V. The maximum voltage corresponds to electric field of 0.37 V nm^−1^ below the voltage breakdown of MgO (>1 V nm^−1^). Figure 1b shows typical differential Kerr images of magnetic DW motion in an annealed CoFeB structure for various gate voltages of *V*_G_=−1.5, 0 and +1.5 V. The dark regions indicate the motion of the DW under a magnetic field pulse of *μ*_0_*H*=8 mT during Δ*t*=45 μs under voltage. The voltage *V*_G_ was applied in the region of the 50 μm-wide ITO strip as indicated by the dashed rectangle. The DW displacement below the ITO strip at *V*_G_=−1.5 V is smaller than that at *V*_G_=0 V, whereas the DW displacement at *V*_G_=1.5 V is larger, indicating an opposite effect of electric field for different polarities. Figure 2a–c shows the dependence of DW velocity on the gate voltage in the annealed sample for different values of *μ*_0_*H*. In all field ranges, the DW velocity increases (decreases) with positive (negative) voltage *V*_G_. As indicated in Figure 2d, the effect of gate voltage on DW velocity is found to be relatively large in the low-field regime, but it strongly decreases with increasing magnetic field. It is noteworthy that these results do not depend on the direction of magnetic fields (Supplementary Fig. 1) and were demonstrated in several devices with different width of ITO electrodes (Supplementary Fig. 2).

**Figure 3:**
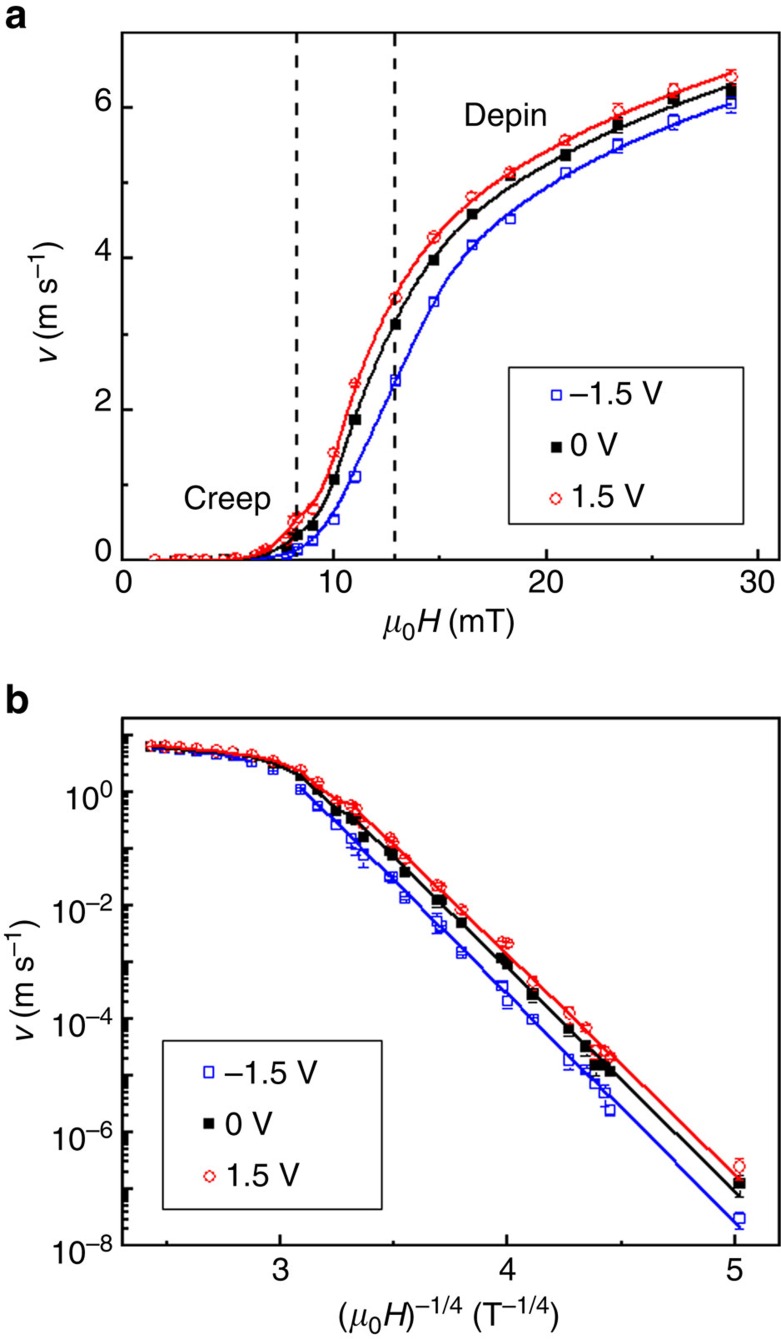
Magnetic DW velocity as a function of the applied magnetic field for different gate voltages in the annealed Ta/CoFeB/MgO structure. (**a**) Velocity as a function of *H* under voltages *V*_G_ of −1.5 V (open squares), 0 V (solid squares) and 1.5 V (open circles). (**b**) Velocity (in logarithmic scale) as a function of *H*^−1/4^ under voltages *V*_G_ of −1.5 V (open squares), 0 V (solid squares) and 1.5 V (open circles). The symbols correspond to the experimental data, and the lines to the fitting (equations (1) and (2)). The error bars of DW velocity are given by repeating the measurements for several times in one device.

**Figure 4:**
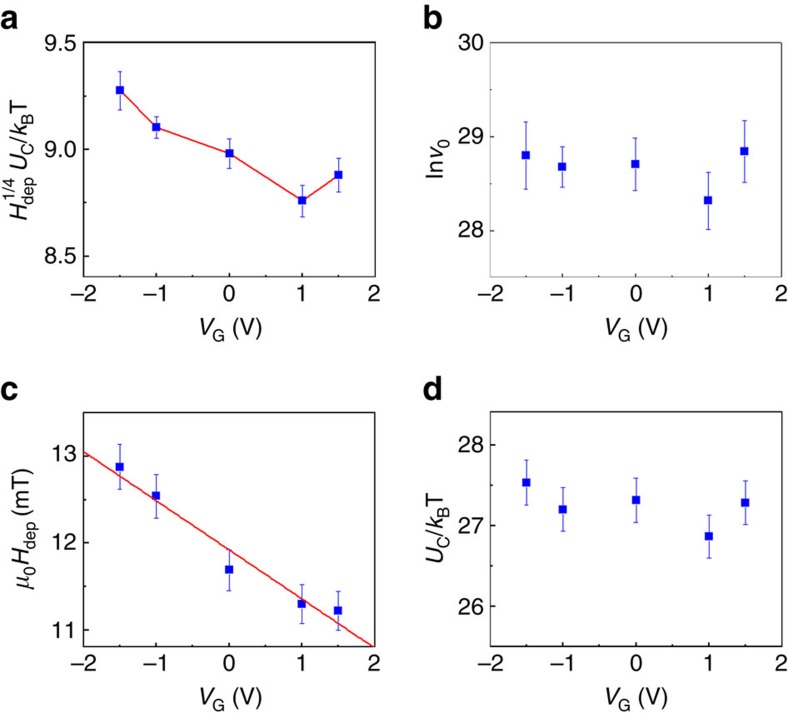
Analysis of the creep and depinning regimes under voltages in the annealed Ta/CoFeB/MgO structure. (**a**–**d**) Variation of *U*_C_(*H*_dep_)^1/4^/(*k*_B_*T*), ln*v*_0_ *μ*_0_*H*_dep_ and *U*_C_/(*k*_B_*T*), respectively, as a function of the gate voltage. *H*_dep_ decreases linearly with increasing *V*_G_. The error bars of the parameters fitting are indicated.

To determine the role of voltage on the various dynamic regimes, we have measured the dependence of DW velocity *v* on the applied magnetic field *H* for various voltages ranging from −1.5 to 1.5 V. Figure 3 shows the DW velocity versus *μ*_0_*H* for typical gate voltages *V*_G_=−1.5, 0 and 1.5 V for the annealed sample. Three DW dynamical regimes are observed, including the creep, intermediate depinning and depinning regimes^11^,^17^,^19^,^20^,^22^. In all regimes the DW velocity increases (decreases) under positive (negative) voltage. In the creep regime where *μ*_0_*H*<8 mT (Fig. 3b), the DW velocity can be expressed as


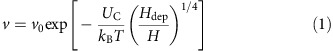


where *U*_C_ is a characteristic energy related to the disorder-induced pinning potential, *k*_B_ the Boltzmann constant, *T* the temperature and *H*_dep_ the depinning field at which the Zeeman energy is equal to the DW pinning energy. The exponent 1/4 fits the data well and is theoretically predicted for interactions of one-dimensional DWs with two-dimensional weak random disorder in thin magnetic films with PMA^11^. This regime allows us to determine the values of *U*_C_(*H*_dep_)^1/4^/(*k*_B_*T*) and ln*v*_0_ as a function of the applied voltage as shown in Figure 4a,b. *U*_C_(*H*_dep_)^1/4^/(*k*_B_*T*) decreases (increases) with positive (negative) voltages values, whereas ln*v*_0_ remains fairly constant. In agreement with ref. 19, close above *H*_dep_ a critical depinning regime is observed for *μ*_0_*H*>12 mT, where the velocity fits as


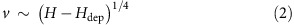


This regime allows us to determine accurately the value of *H*_dep_ as a function of the gate voltages as shown in Fig. 4c. *H*_dep_ decreases with increasing voltage. We find a linear variation of the depinning field with voltage that can be described by


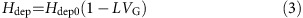


where *H*_dep0_=12 mT and *L*∼0.05 V^−1^. Finally, by using the value of *μ*_0_*H*_dep_ determined from equation (2) into equation (1), the characteristic energy *U*_C_ is found to be independent of the voltage as indicated in Fig. 4d. Although a dependence of *U*_C_ on anisotropy is expected in the creep theory^10^,^11^, this feature is consistent with recent results of modulation of anisotropy in Pt/Co/Pt^32^ and Ta/CoFeB/MgO^33^ films. Between these two regimes for 8 mT<*μ*_0_*H*<12 mT, an intermediate depinning regime occurs, which corresponds to the tails of the creep regime^20^ where the energy barriers vanish linearly as Δ*E*∼(*H*/*H*_dep_−1) approaching *H*_dep_.

### Voltage effect on anisotropy and DW depinning field

Our results are consistent with electric field-induced change of perpendicular anisotropy as described below. In our structure, the dielectric layer consists of a 2 nm-thick MgO (dielectric constant *ɛ*_MgO_∼9.7) and 20 nm-thick TiO_2_ (dielectric constant *ɛ*_TiO2_∼100) films. A positive (negative) gate voltage of *V*_G_=+1 V (−1 V) corresponds to an electric field of ∼0.25 V nm^−1^ and an accumulation (depletion) of ∼0.01 electron per Co(Fe) atom at the CoFeB/MgO interface. The results here show that positive voltage increases the DW velocity. This is consistent with recent studies showing that positive voltage give rise to a reduction of the effective anisotropy *K*_eff_ in Ta/CoFeB/MgO structures^15^,^16^,^34^,^35^,^36^. Indeed, as the depinning field *H*_dep_ depends on *K*_eff_ as *H*_dep_∼(*K*_eff_)^1/2^ (refs 10, 11, 33), a reduction of *K*_eff_ under positive voltage leads to a reduction of *H*_dep_ and thus an increase of DW velocity following equations (1) and (2). It is noteworthy that the variation of *H*_dep_ gives rise to a depinning efficiency of 2.3 mT V^−1^ nm.

In a recent study^36^, we have used ionic liquid to apply electric fields on larger scales in Ta/CoFeB/MgO structures grown under the same conditions, which allows us to measure the variation of the anisotropy field under electric field. The results are found to be consistent with a linear variation of the effective anisotropy under voltage, confirming previous studies^15^,^16^,^34^,^35^. Thus, considering *K*_eff_=*K*_eff0_(1−*aV*_G_), where *K*_eff0_ is the effective anisotropy at zero voltage, *a* is the modulation ratio of *K*_eff_ per V and *V*_G_ the gate voltage, the depinning field *H*_dep_∼(*K*_eff_)^1/2^ can be written as


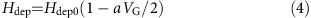


where *H*_dep0_ is the propagation field at zero voltage (Supplementary Note 1). This is consistent with our finding of a linear variation of *H*_dep_ with voltage (see equation (3)) considering *a*/2=*L*. Using *L*=0.05 V^−1^ deduced from the fit to Fig. 4c, we find that the modulation ratio of *K*_eff_ is *a*∼10% per V. As the modulation of *K*_eff_ is related to the modulation of the interface anisotropy at the CoFeB/MgO interface and *K*_eff0_∼10^5^ J m^−3^ in our films, the typical modulation ratio of interface anisotropy *K*_eff_*t*_CoFeB_ where *t*_CoFeB_=1 nm is then 40 fJ m^−1^ V^−1^ in agreement with previous studies on Ta/Co_40_Fe_40_B_20_/MgO (ref. 15).

### Effective magnetic field under voltage

**Figure 5:**
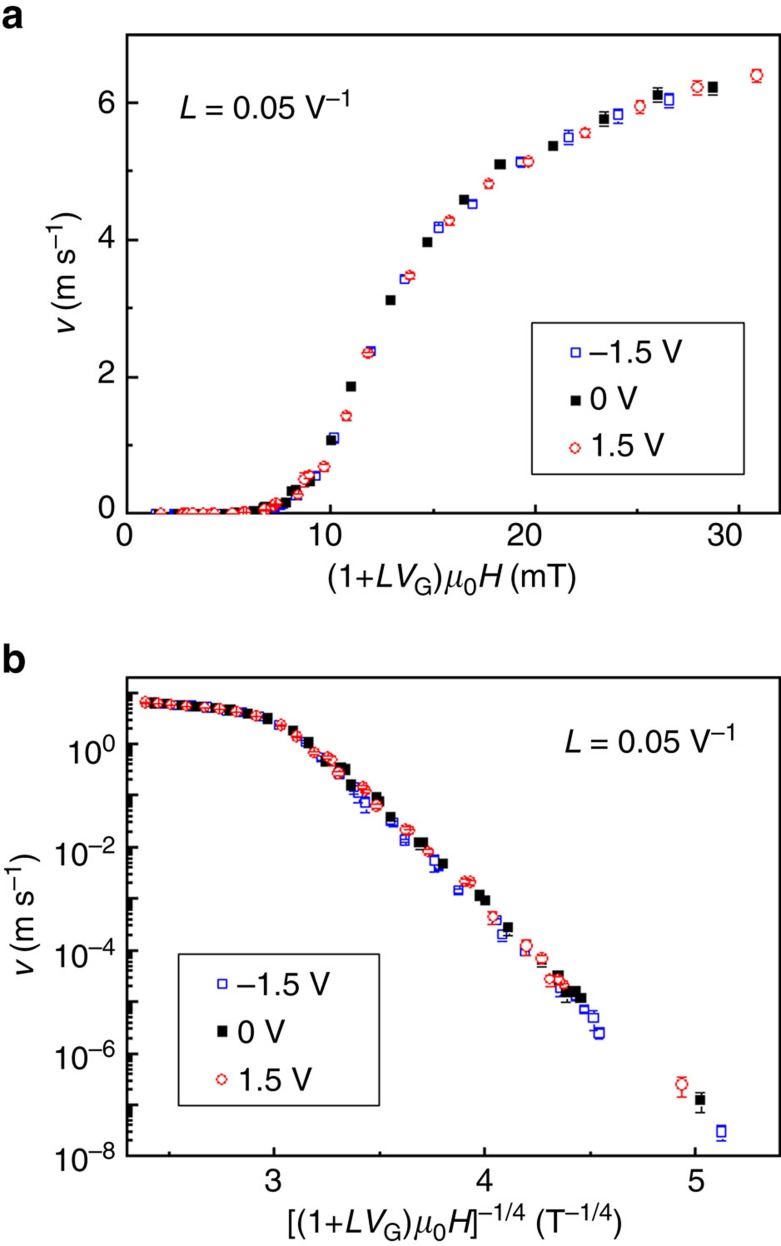
DW velocity as a function of the effective magnetic field *H*_eff_ for different gate voltages in the annealed Ta/CoFeB/MgO structure. (**a**) Velocity *v* as a function of (1+*LV*_G_)*H* under voltages *V*_G_ of −1.5 V (open squares), 0 V (solid squares) and 1.5 V (open circles), where *L*=0.05 V^−1^. (**b**) Velocity *v* (in logarithmic scale) as a function of [(1+*LV*_G_)*H*]^−1/4^ under voltages *V*_G_ of −1.5 V (open squares), 0 V (solid squares) and 1.5 V (open circles), where *L*=0.05 V^−1^. The error bars of DW velocity are given by repeating the measurements for several times in one device.

In the creep and intermediate depinning regime where DW velocities follow an exponential behaviour, the energy barrier depends on *H*/*H*_dep_ that can be written *H*/*H*_dep_=(1+*LV*_G_)*H*/*H*_dep0_. This indicates that the DW velocity under voltage follows the same expressions as the ones at zero voltage by replacing the magnetic field *H* by an effective magnetic field *H*_eff_=(1+*LV*_G_)*H* (Supplementary Fig. 3), that is, the energy barrier is given by *H*_eff_/*H*_dep0_. This is shown in Fig. 5a where the DW velocity *v* is plotted against *H*_eff_ under typical *V*_G_ of −1.5, 0 and 1.5 V, respectively. The behaviour in the DW creep regime is also shown in Fig. 5b where *v* (in logarithmic scale) is plotted against (*H*_eff_)^−1/4^. All the curves can be superimposed by considering the effective magnetic field instead of the applied magnetic field. It is interesting to observe that it is also the case in the depinning regime above *H*_dep_ where the DW velocity depends on (*H*−*H*_dep_)^1/4^. As *LV*_G_<<1, (*H*−*H*_dep_)^1/4^≈(*H*_eff_−*H*_dep0_)^1/4^, which indicates that the universal description given by *H*_eff_ is quantitatively applicable over the full range of the pinning-dependent regimes.

### Voltage effect on magnetic DW velocity in the Walker regime

**Figure 6:**
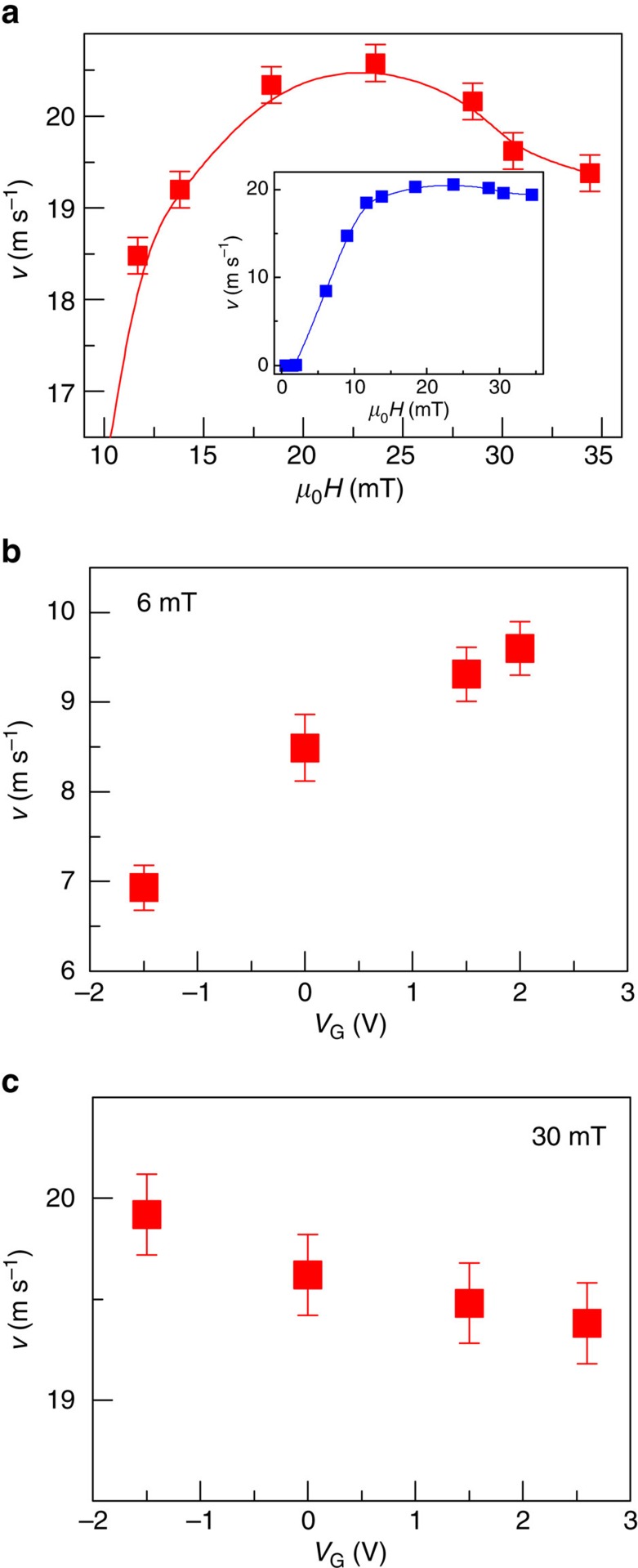
Gate voltage effect on magnetic DW velocity in an as-deposited Ta/CoFeB/MgO structure. (**a**) Velocity *v* as a function of *H* in the range from 11 to 34 mT at zero voltage. Inset shows *v* in the *H* range from 0.6 to 34 mT. (**b**,**c**) Velocity *v* as a function of *V*_G_ under magnetic field *μ*_0_*H* of 6 and 30 mT, respectively. The error bars of DW velocity are given by repeating the measurements for several times in one device.

Figure 6 shows DW velocity at high fields in an as-grown Ta(5 nm)/Co_40_Fe_40_B_20_(1 nm)/MgO(2 nm)/TiO_2_(20 nm)/ITO structure, for which the PMA is lower due to the amorphous character of the CoFeB layer^19^. In this case, the beginning of the flow regime occurs at lower fields than the annealed sample (Supplementary Fig. 4), which allows us to study the influence of electric field on the flow regime. As shown in Fig. 5a, the velocity *v* at *V*_G_=0 as a function of the magnetic field increases up to *μ*_0_*H*=27 mT. Interestingly, for *μ*_0_*H*>27 mT, a new regime where the DW velocity decreases with increasing *H* is observed. This additional regime beyond the depinning regime corresponds to the instability regime above the Walker breakdown that is partially hidden by the depinning regime as we have recently observed^17^,^18^,^21^,^31^. For *μ*_0_*H*<27 mT, the DW velocity increases with positive *V*_G_, as shown in Figure 5b. Interestingly, Fig. 5c shows that for *μ*_0_*H*=30 mT in the negative mobility regime, instead the DW velocity decreases with increasing *V*_G_, indicating a reversal of the electric field effect. In this regime, the DW velocity exhibits a non-trivial dependence on the effective anisotropy. First, we note that the Walker field can be written *H*_W_=*N*_*y*_*M*_*S*_*α*/2, where *N*_y_ is the demagnetizing factor across the wall given as *t*_CoFeB_/(*t*_CoFeB_+Δ), *α* is the damping parameter and Δ is the DW width that can be written Δ=(*A*/*K*_eff_+*N*_y_*μ*_0_*M*_S_^2^/2)^1/2^. The typical value of *H*_W_ is around 0.8 mT in our films^22^ lower than *H*_dep_. As *H*_W_ depends on Δ^−1^, *H*_W_ is expected to vary as (*K*_eff_)^1/2^ such as *H*_dep_. Thus, a positive voltage (reduction of *K*_eff_) results in a decrease of *H*_W_, which, as first approximation, translates the Walker regime to lower fields reducing the DW velocity above *H*_W_ as we observed. This is further supported by micromagnetic simulations (Supplementary Fig. 5), which shows that in the Walker regime indeed a reduction of anisotropy gives rise to a reduction of DW velocity.

## Discussion

In conclusion, beyond the important finding of the universal description of electric field-induced DW motion, the possibility to both obtain depining efficiency up to 2.5 mT V^−1^ nm and control DW velocity up to the flow regime in CoFeB/MgO structures opens new perspectives for low power spintronic applications such as solid state memories and logic devices. We believe that these electric field effects can be used to lower the energy barrier for stored DWs, leading to a smaller spin-polarized currents to depin and move them. In addition, as the depinning fields of CoFeB/MgO can be as small as 2 mT, on/off operations are feasible, which is of interest for logic devices. Furthermore, electric field effects can be very useful in the flow regime to speed up DW motion between two storing positions. The control of DW motion is obtained through interface anisotropy modulation due to charge accumulation/depletion at the ferromagnetic metal/dielectric interfaces. To overcome the limited charge modulation related to the static breakdown of the dielectric layer, pulsed gate voltages together with optimized dielectrics layers can provide much larger electric field, which could be an avenue for higher efficiencies.

## Methods

### Fabrication of Ta/CoFeB/MgO/TiO_2_/ITO structure

The Ta(5 nm)/Co_40_Fe_40_B_20_(1 nm)/MgO(2 nm)/Ta(5 nm) films were deposited on Si/SiO_2_ wafers by magnetron sputtering. Atomic force microscopy and X-ray reflectivity indicate high structural quality with very low interface roughness^37^,^38^. Selected films were annealed at 300 °C for 2 h. To apply an electric field effect in CoFeB/MgO structures, a high dielectric constant layer is required to be deposited on top of the MgO layer. The top 5 nm-thick Ta layer was gently removed using ion beam etching monitored by secondary ion beam spectroscopy, and then a 20 nm-thick TiO_2_ dielectric layer was sputtered on the MgO layer from a Ti target with mixed Ar and O_2_ gas (Supplementary Fig. 6). Finally, 100 nm-thick sputtered ITO (In_2_O_3_:Sn) stripes with width ranging from 10 to 50 μm were patterned on top of the TiO_2_ layer as the top electrode by optical lithography and lift-off process. ITO was used as the top electrode, because it is not only a good electrical conductor (conductivity∼7 Ω^−1^ m^−1^), but also is transparent for visible light, so that it is possible to use Kerr microscopy for visualizing magnetic domains. By applying a top gate voltage *V*_G_ between the bottom Ta/CoFeB and the top ITO, electrons can accumulate or deplete at the CoFeB/MgO interface. Here, positive (negative) gate voltage corresponds to electron accumulation (depletion) in the CoFeB layer. The leakage current was measured to be less than a few nA for gate voltages *V*_G_<1.5 V and <100 nA at the maximum gate voltage (Supplementary Fig. 6), and thus Joule heating influence on DW motion is negligible.

### Magnetic DW velocity measurement by polar Kerr microscopy

The polar Kerr microscopy was performed using a diode with the blue light (455 nm in wavelength) and a × 50 objective lens. The analyser was set to ∼87° (analyser and polarizer almost crossed) and the exposure time was 2 s. To measure DW velocity under an applied perpendicular magnetic field *H*, we used the following procedures: first, the sample was saturated with a magnetic field of −20 mT fixed for >10 s. Second, several positive magnetic field pulses of +3 mT with duration of around 10 s were applied, until the nucleation of a single DW below the ITO strip was observed. A reference Kerr image was taken at zero applied magnetic field after the first set of magnetic field pulses. Third, another magnetic field pulse was applied, resulting in a DW displacement. The amplitude of the second set of magnetic field pulses ranged from 0.6 to 35 mT and their durations Δ*t* from 100 s (long pulse) to 9 μs (short pulse). The magnetic field pulse was generated from a coil with 1 cm diameter, which was fixed very close to the sample, and the rise time of magnetic field was ∼4 μs. After the second set of magnetic field pulses were applied, a second Kerr image was taken as soon as the magnetic field was back to zero. The DW displacement during Δ*t* was obtained by the difference between the two Kerr images, which gave the DW velocity *v*. The velocity measured under the short magnetic field pulses was determined by the slope of the DW displacement versus magnetic field pulse duration.

### Data availability

The data that support the findings of this study are available from the corresponding author upon request.

## Additional information

**How to cite this article**: Lin, W. *et al*. Universal domain wall dynamics under electric field in Ta/CoFeB/MgO devices with perpendicular anisotropy. *Nat. Commun.* **7**, 13532 doi: 10.1038/ncomms13532 (2016).

**Publisher's note:** Springer Nature remains neutral with regard to jurisdictional claims in published maps and institutional affiliations.

## Supplementary Material

Supplementary Information Supplementary Figures 1-6, Supplementary Note 1, Supplementary References.

## References

[b1] Yamanouchi M., Chiba D., Matsukura F. & Ohno H. Current-assisted domain wall motion in ferromagnetic semiconductors. Jpn J. Appl. Phys. 45, 3854–3859 (2006).

[b2] Logginov A. S. . Room temperature magnetoelectric control of micromagnetic structure in iron garnet films. Appl. Phys. Lett. 93, 182510 (2008).

[b3] Schellekens A. J., van den Brink A., Franken J. H., Swagten H. J. M. & Koopmans B. Electric-field control of domain wall motion in perpendicularly magnetized materials. Nat. Commun. 3, 847 (2012). 22617287 10.1038/ncomms1848

[b4] Chiba D. . Electric-field control of magnetic domain-wall velocity in ultrathin cobalt with perpendicular magnetization. Nat. Commun. 3, 888 (2012). 22673915 10.1038/ncomms1888

[b5] Bauer U., Emori S. & Beach G. S. D. Voltage-gated modulation of domain wall creep dynamics in an ultrathin metallic ferromagnet. Appl. Phys. Lett. 101, 172403 (2012).

[b6] Bernand-Mantel A. . Electric-field control of domain wall nucleation and pinning in a metallic ferromagnet. Appl. Phys. Lett. 102, 122406 (2013).

[b7] Bauer U., Emori S. & Beach G. S. D. Voltage-controlled domain wall traps in ferromagnetic nanowires. Nat. Nanotechnol. 8, 411–416 (2013). 23708429 10.1038/nnano.2013.96

[b8] Parkin S. S. P., Hayashi M. & Thomas L. Magnetic domain-wall racetrack memory. Science 320, 190–194 (2008). 18403702 10.1126/science.1145799

[b9] Allwood D. A. . Submicrometer ferromagnetic NOT gate and shift register. Science 296, 2003–2006 (2002). 12065830 10.1126/science.1070595

[b10] Ferré J. in Spin Dynamics in Confined Magnetic Structures I eds Hillebrands B., Ounadjela K.) Vol. 83, p 127–168Springer-Verlag: Berlin Heidelberg, (2002).

[b11] Lemerle S. . Domain wall creep in an Ising ultrathin magnetic film. Phys. Rev. Lett. 80, 849–852 (1998).

[b12] Weisheit M. . Electric field-induced modification of magnetism in thin-film ferromagnets. Science 315, 349–351 (2007). 17234941 10.1126/science.1136629

[b13] Maruyama T. . Large voltage-induced magnetic anisotropy change in a few atomic layers of iron. Nat. Nanotechnol. 4, 158–161 (2009). 19265844 10.1038/nnano.2008.406

[b14] Niranjan M. K., Duan C.-G., Jaswal S. S. & Tsymbal E. Y. Electric field effect on magnetization at the Fe/MgO(001) interface. Appl. Phys. Lett. 96, 222504 (2010).

[b15] Endo M. . Electric-field effects on thickness dependent magnetic anisotropy of sputtered MgO/Co_40_Fe_40_B_20_/Ta structures. Appl. Phys. Lett. 96, 212503 (2010).

[b16] Kita K., Abraham D. W., Gajek M. J. & Worledge D. C. Electric-field-control of magnetic anisotropy of Co_0.6_Fe_0.2_B_0.2_/oxide stacks using reduced voltage. J. Appl. Phys. 112, 033919 (2012).

[b17] Metaxas P. J. . Creep and flow regimes of magnetic domain-wall motion in ultrathin Pt/Co/Pt films with perpendicular anisotropy. Phys. Rev. Lett. 99, 217208 (2007). 18233251 10.1103/PhysRevLett.99.217208

[b18] Yamada K. . Influence of instabilities on high-field magnetic domain wall velocity in (Co/Ni) nanostrips. Appl. Phys. Express 4, 113001 (2011).

[b19] Gorchon J. . Pinning-dependent field-driven domain wall dynamics and thermal scaling in an ultrathin Pt/Co/Pt magnetic film. Phys. Rev. Lett. 113, 027205 (2014). 25062227 10.1103/PhysRevLett.113.027205

[b20] Jeudy V. . Universal pinning energy barrier for driven domain walls in thin ferromagnetic films. Phys. Rev. Lett. 117, 057201 (2016). 27517790 10.1103/PhysRevLett.117.057201

[b21] Devolder T. . Damping of Co_x_Fe_80-x_B_20_ ultrathin films with perpendicular magnetic anisotropy. Appl. Phys. Lett. 102, 022407 (2013).

[b22] Burrowes C. . Low depinning fields in Ta-CoFeB-MgO ultrathin films with perpendicular magnetic anisotropy. Appl. Phys. Lett. 103, 182401 (2013).

[b23] Tetienne J.-P. . Nanosacle imaging and control of domain wall hoping with a nitrogen vacancy center microscope. Science 344, 1366–1369 (2014). 24948732 10.1126/science.1250113

[b24] Mizukami S. . Gilbert damping in perpendicularly magnetized Pt/Co/Pt films investigated by all-optical pump-probe technique. Appl. Phys. Lett. 96, 152502 (2010).

[b25] Mizukami S. . Gilbert damping in Ni/Co multilayer films exhibiting large perpendicular anisotropy. Appl. Phys. Express 4, 013005 (2011).

[b26] Ikeda S. . A perpendicular-anisotropy CoFeB-MgO magnetic tunnel junction. Nat. Mater. 9, 721–724 (2010). 20622862 10.1038/nmat2804

[b27] Worledge D. C. . Spin torque switching of perpendicular Ta/CoFeB/MgO-based magnetic tunnel junctions. Appl. Phys. Lett. 98, 022501 (2011).

[b28] Fukami S. . Current-induced domain wall motion in perpendicularly magnetized CoFeB nanowire. Appl. Phys. Lett. 98, 082504 (2011).

[b29] Thiaville A., Rohart S., Jué E., Cros V. & Fert A. Dynamics of Dzyaloshinskii domain walls in ultrathin magnetic films. Europhys. Lett. 100, 57002 (2012).

[b30] Ryu K.-S., Thomas L., Yang S.-H. & Parkin S. Chiral spin torque at magnetic domain walls. Nat. Nanotechnol. 8, 527–533 (2013). 23770808 10.1038/nnano.2013.102

[b31] Emori S., Bauer U., Ahn S.-M., Martinez E. & Beach G. S. D. Current-driven dynamics of chiral ferromagnetic domain walls. Nat. Mater. 12, 611–616 (2013). 23770726 10.1038/nmat3675

[b32] Shepley P. M. . Modification of perpendicular magneticanisotropy and domain wall velocity in Pt/Co/Pt by voltage-induced strain. Sci. Rep. 5, 7921 (2015). 25605499 10.1038/srep07921 PMC4300497

[b33] Herrera Diez L. . Controlling magnetic domain wall motion in the creep regime in He^+^-irradiated CoFeB/MgO films with perpendicular anisotropy. Appl. Phys. Lett. 107, 032401 (2015).

[b34] Wang W. G., Li M., Hageman S. & Chien C. L. Electric-field-assisted switching in magnetic tunnel junctions. Nat. Mater. 11, 64–68 (2012). 10.1038/nmat3171 22081084

[b35] Kanai S., Martin Gajek M., Worledge M., Matsukura F. & Ohno H. Electric field-induced ferromagnetic resonance in a CoFeB/MgO magnetic tunnel junction under dc bias voltages. Appl. Phys. Lett. 105, 242409 (2014).

[b36] Liu Y. T. . Ionic-liquid gating of perpendicularly magnetised CoFeB/MgO thin films. J. Appl. Phys. 120, 023901 (2016).

[b37] Mantovan R. . Perpendicular magnetic anisotropy in Ta/CoFeB/MgO systems synthesized on treated SiN/SiO_2_ substrates for magnetic memories. Thin Solid Films 533, 75–78 (2013).

[b38] Lamperti A., Ahn S.-M., Ocker B., Mantovan R. & Ravelosona D. Interface width evaluation in thin layered CoFeB/MgO multilayers including Ru or Ta buffer layer by X-ray reflectivity. Thin Solid Films 533, 79–82 (2013).

