# Supplementary material for: Universal domain wall dynamics under electric field in Ta/CoFeB/MgO devices with perpendicular anisotropy
Source: Nat Commun. 2016 Nov 16;7:13532. doi: 10.1038/ncomms13532 (PMC5116098; doi:10.1038/ncomms13532)
Supplement: Supplementary Information — Supplementary Figures 1-6, Supplementary Note 1, Supplementary References. [file ncomms13532-s1.pdf]

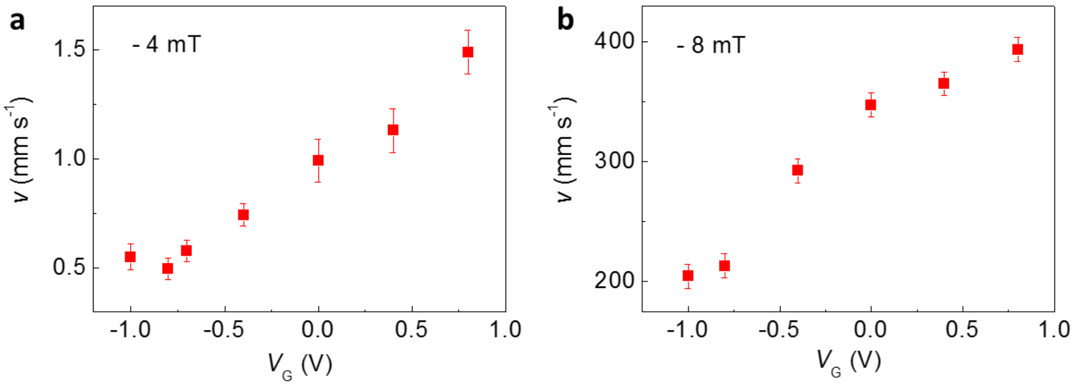

Supplementary Figure 1 | Gate voltage dependence of DW velocity under negative applied magnetic fields in the annealed Ta(5 nm)/$Co_{40}Fe_{40}B_{20}$(1 nm)/MgO(2 nm)/TiO_2(20 nm)/ITO device. (**a**) $\mu_0 H$ = -4 mT. (**b**) $\mu_0 H$ = -8 mT. The gate voltage dependence V_G of DW velocity v under negative applied magnetic fields shows the same trend as positive magnetic fields (shown in Fig. 2 of the main text). The error bars of DW velocity are measured by repeating the measurements several times in each device.

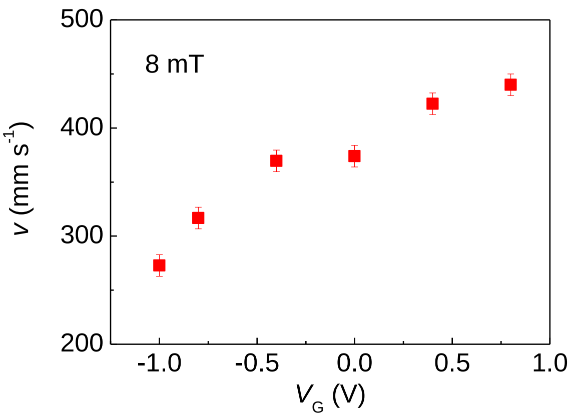

Supplementary Figure 2 | Gate voltage dependence of DW velocity in the annealed Ta/$Co_{40}Fe_{40}B_{20}$/MgO/TiO_2 device with a 20 µm wide ITO electrode for an applied magnetic field of 8 mT. The gate voltage dependence of DW velocity in the annealed Ta/$Co_{40}Fe_{40}B_{20}$/MgO/TiO_2 device with a 20 µm wide ITO electrode exhibits similar behavior than that with 50 µm wide ITO electrode (shown in Fig. 2 of the main text). The only difference is the absolute value of DW velocity at a given magnetic field, which is strongly dependent on intrinsic disorder and defects introduced by the nanotechnology process and vary from one device to another. The error bars of DW velocity are measured by repeating the measurements several times in each device.

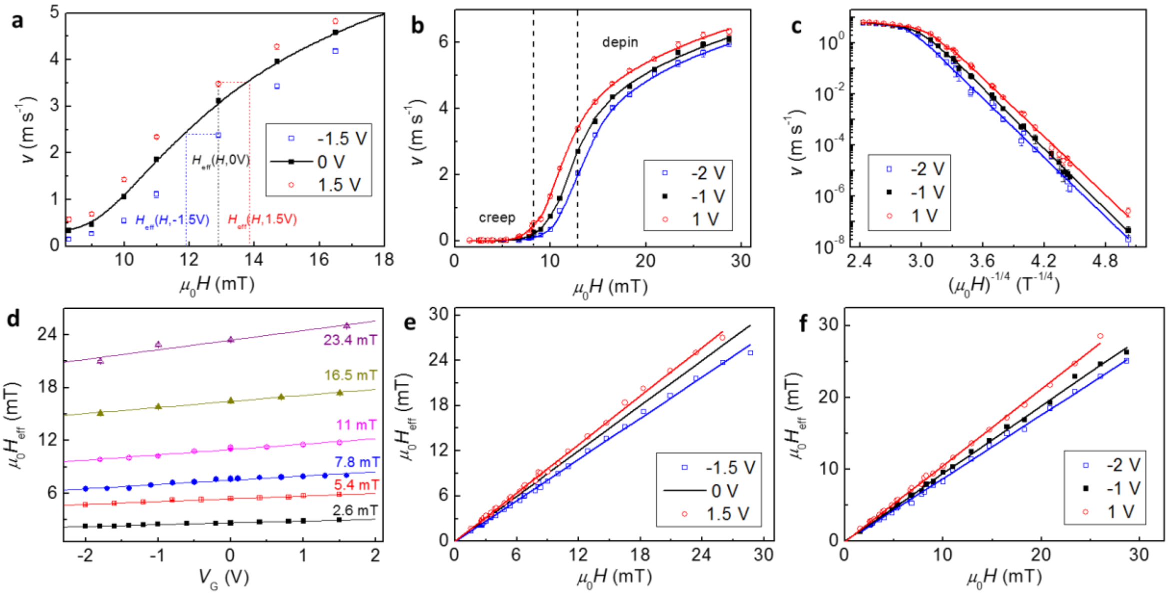

Supplementary Figure 3 | Effective magnetic field under voltage. (**a**) Determination of the effective magnetic field H_{eff} under gate voltage V_G in the annealed Ta/$Co_{40}Fe_{40}B_{20}$/MgO/TiO_2 device. Since DW velocities depend on both applied magnetic field and gate voltage, we can define an effective magnetic field H_{eff} where $v(H, V_G) = v(H_{eff}, V_G = 0)$. For instance, $v(\mu_0 H = 13$ mT, $V_G = -1.5$ V) corresponds to $v(\mu_0 H_{eff} = 11.8$ mT, $V_G = 0$ V). Thus the effect of a −1.5 V voltage is equivalent to reducing the magnetic field by about 1.2 mT. (**b**) DW velocity v as a function of H under V_G of −2 V (open squares), −1 V (solid squares) and 1 V (open circles) in the annealed Ta/$Co_{40}Fe_{40}B_{20}$/MgO/TiO_2 device. (**c**) DW velocity v (in logarithmic scale) as a function of $H^{-1/4}$ under voltages V_G of −2 V (open squares), −1 V (solid squares) and 1 V (open circles). (**d**) H_{eff} as a function of V_G for various H. The linear fittings of H_{eff} vs V_G curves are shown for each field H. (**e**) H_{eff} as a function of H under V_G of −1.5 V (open squares) and 1.5 V (open circles). The linear fittings of H_{eff} vs H curves are shown for different voltages. (**f**) H_{eff} as a function of H under gate voltages V_G of −2 V, −1 V and 1 V. The linear fittings of H_{eff} vs H curves are shown for different voltages. H_{eff} is found to be linear with V_G for various applied field H and proportional to H for various V_G. Based on this analysis, we find that H_{eff} can be written as $H_{eff}(V_G, H) = (1 + LV_G)H$, where the coefficient L is 0.050 ± 0.005 V^{-1} for this device. The error bars of DW velocity are given by repeating the measurements for several times in one device.

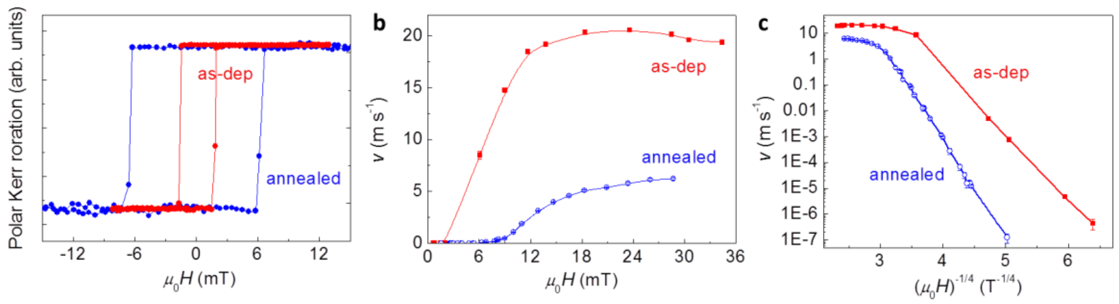

Supplementary Figure 4 | Magnetic properties and DW velocity in as-grown and annealed Ta(5 nm)/$Co_{40}Fe_{40}B_{20}$(1 nm)/MgO(2 nm)/TiO_2(20 nm) films. (**a**) Polar Kerr loops of the as-grown (blue) and annealed (red) films. The coercivity of the annealed Ta/$Co_{40}Fe_{40}B_{20}$/MgO/TiO_2 film is larger than the as-grown one. (**b**) DW velocity as a function of H for the as-grown and annealed films. (**c**) DW velocity (in logarithmic scale) as a function of $H^{-1/4}$. The error bars of DW velocity are given by repeating the measurements several times in each sample. At a given magnetic field, the DW velocity for the as-grown film is faster than that of the annealed one. The DW depinning fields are 6.1 mT and 11.7 mT for the as-grown Ta/$Co_{40}Fe_{40}B_{20}$/MgO/TiO_2 film and

the annealed one, respectively. By fitting the curve ln*v* vs $H^{-1/4}$ curve in the creep regime, as shown in (**c**), the pinning barriers U_C are 8.54 × 10^{-20} J and 1.12 × 10^{-19} J for the as-grown Ta/$Co_{40}Fe_{40}B_{20}$/MgO/TiO_2 film and the annealed one, respectively. These results may be related to more defects in the film after annealing as we have shown recently[1]. As a result of lower propagation fields in As-grown samples, the negative mobility of the Walker regime is observable.

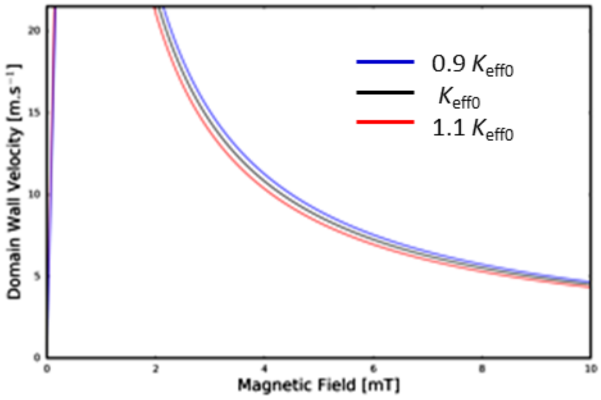

Supplementary Figure 5 | Micromagnetic simulations of DW velocity in the flow (Walker) regime vs *H* using the 1D model in $Co_{40}Fe_{40}B_{20}$ films for ±10% variation of K_{eff}. K_{eff} = 0.9K_{eff0} (red), K_{eff0} (black), 1.1K_{eff0} (blue), respectively. In the Walker regime with negative mobility, a reduction of magnetic anisotropy leads to a decrease of DW velocity.

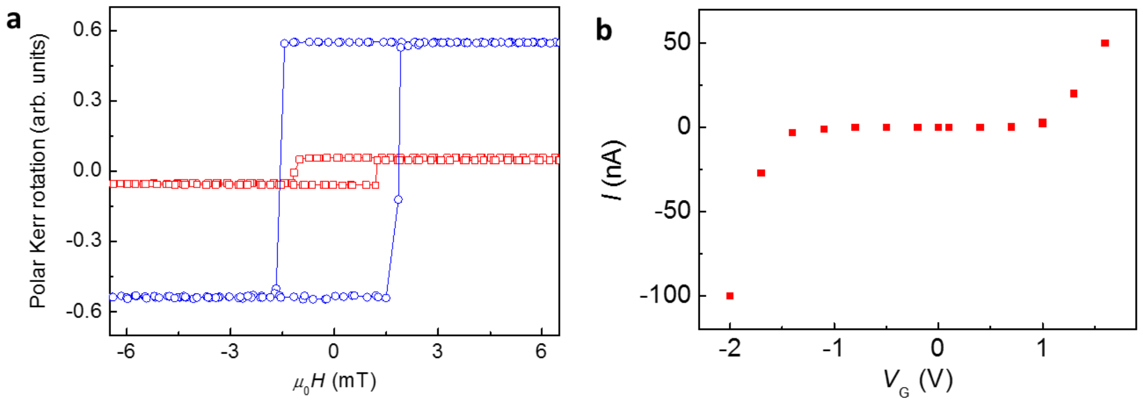

Supplementary Figure 6 | Fabrication process influence on magnetic properties of the Ta/CoFeB/MgO films and leakage current in the Ta/$Co_{40}Fe_{40}B_{20}$/MgO/TiO_2/ITO device. (**a**) Polar Kerr loops of annealed Ta(5 nm)/$Co_{40}Fe_{40}B_{20}$(1 nm)/MgO(2 nm)/Ta(5 nm) (red squares) and Ta(5 nm)/$Co_{40}Fe_{40}B_{20}$(1 nm)/MgO(2 nm)/TiO_2(20 nm) films (blue circles). The annealed Ta/$Co_{40}Fe_{40}B_{20}$/MgO/TiO_2 film exhibits a hysteresis loop with a larger coercivity and Kerr rotation than that of the annealed Ta/$Co_{40}Fe_{40}B_{20}$/MgO/Ta film. This is probably related to the change of oxidation of the MgO barrier during the process of the Ta etching and the TiO_2 deposition. As a consequence, the depinning field in the Ta/$Co_{40}Fe_{40}B_{20}$/MgO/TiO_2 film is slightly higher than that in the Ta/$Co_{40}Fe_{40}B_{20}$/MgO/Ta film indicating more pinning defects probably related to the CoFeB/MgO interface. (**b**) Gate dependence of leakage current in the annealed Ta/$Co_{40}Fe_{40}B_{20}$/MgO/TiO_2/ITO device. The leakage current in our samples is very low (< 50 nA for -1.5 V < V_G < 1.5 V).

Supplementary Note 1. Voltage induced modulation of magnetic anisotropy and DW

depinning field

In Ta/CoFeB/MgO structures, the effective magnetic anisotropy can be written $K_{\mathrm{eff}} = \frac{K_{\mathrm{S}}}{t_{\mathrm{CoFeB}}} - 2\pi M_S^2$, where K_{S} is the interface magnetic anisotropy, t_{CoFeB} the thickness and M_{S} the saturation magnetization. It has been found that K_{S} varies linearly with the gate voltage V_{G} (Refs. 2 and 3), which can be expressed as $K_{\mathrm{S}} = K_{\mathrm{S0}} - AV_{\mathrm{G}}$. Then, $K_{\mathrm{eff}} = \frac{K_{\mathrm{S0}} - AV_{\mathrm{G}}}{t_{\mathrm{CoFeB}}} - 2\pi M_S^2$. Since $K_{\mathrm{eff0}} = \frac{K_{\mathrm{S0}}}{t_{\mathrm{CoFeB}}} - 2\pi M_S^2$, we can write $K_{\mathrm{eff}} = K_{\mathrm{eff0}} - \frac{AV_{\mathrm{G}}}{t_{\mathrm{CoFeB}}} = K_{\mathrm{eff0}}\left(1 - \frac{AV_{\mathrm{G}}}{K_{\mathrm{eff0}} t_{\mathrm{CoFeB}}}\right)$. Thus the gate voltage dependence of the effective magnetic anisotropy can be written as $K_{\mathrm{eff}} = K_{\mathrm{eff0}}(1 - aV_{\mathrm{G}})$, where $a = \frac{A}{K_{\mathrm{eff0}} t_{\mathrm{CoFeB}}}$.

In the thermally activated regimes, $H_{\mathrm{dep}} \propto (K_{\mathrm{eff}})^{\frac{1}{2}} = (K_{\mathrm{eff0}})^{1/2}(1 - aV_{\mathrm{G}})^{1/2}$ and $H_{\mathrm{dep0}} \propto (K_{\mathrm{eff0}})^{1/2}$. We then can write $H_{\mathrm{dep}} = H_{\mathrm{dep0}}(1 - aV_{\mathrm{G}})^{1/2} \approx H_{\mathrm{dep0}}(1 - \frac{a}{2}V_{\mathrm{G}})$.

Supplementary References:

1. Burrowes, C. *et al.* Low depinning fields in Ta-CoFeB-MgO ultrathin films with perpendicular magnetic anisotropy. *Appl. Phys. Lett.* **103**, 182401 (2013).
2. Endo, M. *et al.* Electric-field effects on thickness dependent magnetic anisotropy of sputtered MgO/$Co_{40}Fe_{40}B_{20}$/Ta structures. *Appl. Phys. Lett.* **96**, 212503 (2010).
3. Kita, K., Abraham, D. W., Gajek, M. J. & Worledge, D. C. Electric-field-control of magnetic anisotropy of $Co_{0.6}Fe_{0.2}B_{0.2}$/oxide stacks using reduced voltage. *J. Appl. Phys.* **112**, 033919 (2012).
